# The College of Nursing. (Limited by Guarantee)

**Published:** 1920-12-11

**Authors:** 


### The College of Nursing.

**(Limited by Guarantee.)**

BIRMINGHAM AND THREE COUNTIES CENTRE.

On Tuesday, December 7, at 5.30 p.m., in the Lecture Theatre of the General Hospital, Birmingham (by kind permission of the Governors), under the presidency of Miss Musson, R.R.C., Mr. Screwby, Divisional Officer for the Ministry of Labour, explained the provisions of the Unemployment Insurance Act, 1920.

Mr. Screwby explained that a pronouncement will be made in a few days by the Minister of Labour as to whether or not nurses are to be included in the Act. In the course of his address he made clear the following points: The 1920 Unemployment Act repeals all previous Unemployment Acts; it applies to all those who are insurable under the Health Insurance Act with one or two exceptions—viz., agricultural labourers, domestic servants, and groups of industries possessing certain classifications amongst which the nursing profession is not included. The unemployment tax is collected by means of stamps affixed to books, which must be lodged, before benefit can be claimed, at a Labour Exchange. The limit of benefit in one calendar year is fifteen weeks; the applicant must show proof of unemployment, and sign the book daily at the Labour Exchange.

An industry may run a special scheme under certain conditions—viz., the scheme must apply to the whole industry, and must provide benefits equivalent to the State scheme.

A vote of thanks was unanimously accorded to the speaker at the close of his address.

The College members discussed the Act, and a resolution was passed unanimously in favour of the exclusion of nurses from the Act, but if this was impossible, their inclusion in a special scheme. A further resolution was also passed that the meeting was of opinion that a statutory superannuation scheme should be initiated for nurses.

Finally, a vote of thanks was accorded to Lord and Lady Cowdray for their gift of 20 Cavendish Square as a Club and Headquarters for the College of Nursing.

